# An Automated Method for High-Throughput Screening of *Arabidopsis* Rosette Growth in Multi-Well Plates and Its Validation in Stress Conditions

**DOI:** 10.3389/fpls.2017.01702

**Published:** 2017-10-04

**Authors:** Nuria De Diego, Tomáš Fürst, Jan F. Humplík, Lydia Ugena, Kateřina Podlešáková, Lukáš Spíchal

**Affiliations:** ^1^Department of Chemical Biology and Genetics, Centre of the Region Haná for Biotechnological and Agricultural Research, Faculty of Science, Palacký University, Olomouc, Czechia; ^2^Laboratory of Growth Regulators, Centre of the Region Haná for Biotechnological and Agricultural Research, Institute of Experimental Botany, Czech Academy of Sciences, Olomouc, Czechia

**Keywords:** high-throughput screening assay, *Arabidopsis*, multi-well plates, rosette growth, stress conditions

## Abstract

High-throughput plant phenotyping platforms provide new possibilities for automated, fast scoring of several plant growth and development traits, followed over time using non-invasive sensors. Using *Arabidops*is as a model offers important advantages for high-throughput screening with the opportunity to extrapolate the results obtained to other crops of commercial interest. In this study we describe the development of a highly reproducible high-throughput *Arabidopsis in vitro* bioassay established using our OloPhen platform, suitable for analysis of rosette growth in multi-well plates. This method was successfully validated on example of multivariate analysis of *Arabidopsis* rosette growth in different salt concentrations and the interaction with varying nutritional composition of the growth medium. Several traits such as changes in the rosette area, relative growth rate, survival rate and homogeneity of the population are scored using fully automated RGB imaging and subsequent image analysis. The assay can be used for fast screening of the biological activity of chemical libraries, phenotypes of transgenic or recombinant inbred lines, or to search for potential quantitative trait loci. It is especially valuable for selecting genotypes or growth conditions that improve plant stress tolerance.

## Introduction

Large-scale plant phenotyping has become an important tool in plant biology and agriculture and contributes significantly to cutting-edge plant breeding and management approaches needed to meet future food and fuel demands. However, the application of high-throughput approaches is still severely limited by a lack of appropriate instrumentation and experimental standards, which would allow better communication of the experimental results and outcomes of any analyses. Identifying good practices associated with performing high-throughput phenotyping of large plant populations is a current challenge for achieving high genotyping capacity and expanding our knowledge of plant development in different environments (Humplík et al., 2015a; Rousseau et al., 2015). In this context, the use of non-invasive imaging techniques has potential for revealing morphological and physiological traits related to plant responses, such as growth. Usually, this trait is described as biomass formation, determined as the weight of the whole plant or part of it (most often the shoots) at a given point in its lifespan. However, classical biomass determination involves the destruction of the plant thus allowing only end-point analysis; this means that the developmental course (kinetics) of the single organ cannot be monitored. To address this, many phenotyping platforms take advantage of relatively simple red–green-blue (RGB) imaging and subsequent software image analysis for non-destructive assessment of the growth of intact plants (Skirycz et al., 2011; Rahaman et al., 2015). Besides, new integrated analysis platform has been also designed combining imaging data analysis obtained from different spectra (Klukas et al., 2014; for review see Humplík et al., 2015b).

Non-invasive techniques for plant growth determination have demonstrated high correlations between the projected area and the biomass, expressed as fresh or dry weight of the shoot, in many plant species including *Arabidopsis*. Although without agronomic significance, *Arabidopsis* offers important advantages for high-throughput screening (HTS). It is a small plant, well-characterized in terms of growth-regulating molecular mechanisms, making this species highly suitable for phenotypic analysis. In addition, new studies have demonstrated the possibility of extrapolating results obtained for *Arabidopsis* using HTS methods to other crops that are of commercial interest, such as tomato, lettuce, carrots, etc. (Rodriguez-Furlán et al., 2016). However, there are still limitations to the actual automated phenotyping methodologies for *Arabidopsis*. In recent years, the development of new techniques has allowed an increase in the number of plants in an experiment: from 3–6 plants per treatment in manual phenotyping studies (Mishra et al., 2014) to 200–1000 plants per whole experiment, depending on the level of automation, platform capacity and the number of variants (Vasseur et al., 2014; Flood et al., 2016). Thus, the maximum number of experimental variants per experiment, e.g., the number of simultaneously studied growth conditions, is determined by the number of plants per variant and the number of technical replicates of each variant. Recently, new methods using semi-automated systems of image acquisition by microscope or scanner for scoring *Arabidopsis* growth *in vitro* in 15 cm Petri dishes and 24-well plates, respectively, were published, allowing an increase in the number of plants per treatment and number of replicates (Rodriguez-Furlán et al., 2016; Tomé et al., 2017).

Several potential complications and methodological difficulties have been identified in some phenotyping platforms; these included spatial and temporal variability of micrometeorological conditions within a growth chamber, differences in soil moisture maintenance, and plant growth capacity after sowing (Granier et al., 2006). Thus, the real HTS of a phenotyping platform is highly dependent on the experimental design selected, which needs to be precisely optimized and standardized to minimize the number of variables influencing the accuracy and reproducibility of the procedure. Methods to improve image acquisition and recommendations for data handling can be found in the literature (e.g., Li et al., 2014; Krajewski et al., 2015). Nevertheless, despite the fact that the correct experimental setup, including selection of the plant material, has significant influence on the success of automated high-throughput phenotyping, there is little published information explaining its relevance.

In the work presented here, we report on the development and optimization of growing protocol suitable for HTS of *Arabidopsis* rosette growth in multi-well plates under salinity as plant stress condition. This approach will allow simultaneous testing of a large number of potentially bioactive compounds in a wide range of concentrations and/or genotypes, under various growth conditions. The relevance of choosing the appropriate experimental design is emphasized and examples illustrating its importance are presented for each case studied and then discussed.

## Materials and Methods

### Plant Material and Growth Conditions

*Arabidopsis thaliana* (accession Col-0) was used in all experiments. Seeds were surface-sterilized, sown on square plates (12 cm × 12 cm) containing 0.5× Murashige-Skoog (MS) medium (Murashige and Skoog, 1962) (pH 5.7) supplemented with a gelling agent 0.6% Phytagel (Sigma–Aldrich, Germany) and maintained for 3 days at 4°C in the dark. Thereafter, the plates were transferred into a growth-chamber with controlled conditions (22°C, 16/8 h light/dark cycle, a photon irradiance of 120 μmol photons of PAR m^-2^ s^-1^) and placed in a vertical position. Three days after germination, seedlings of similar size were transferred under sterile conditions into the multi-well plates [12- and 24-well plates (Jetbiofil, Guangzhou, China)] one seedling per well and the plates were sealed with perforated transparent foil allowing gas and water exchange. Each well contained 2.7 mL (12-well plate) or 1.3 mL (24-well plate) of full MS medium (pH 5.7; supplemented with 0.6% Phytagel). For optimization, different concentrations of MS (1×, 0.5×, and 0.25×) and sucrose (0, 0.1, and 1%) (pH 5.7; containing 0.6% Phytagel) were also used. In the salt-stress experiment 12- and 24-well plates were used filled with 1× MS medium (pH 5.7; containing 0.6% Phytagel) with the addition of NaCl to achieve specific salinities (50, 75, 100, and 150 mM NaCl). In the experiment dealing with interacting growth conditions, 12-well plates containing different MS concentrations (1×, 0.5×, and 0.25×) with or without salt stress (75 mM NaCl) were used.

### Phenotyping Platform, Experimental Setup and Assay Conditions

**FIGURE 1:**
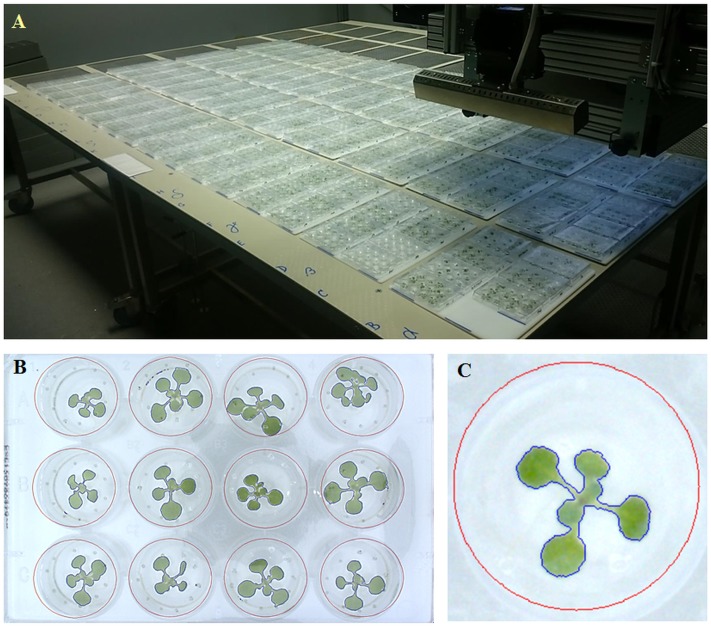
High-throughput phenotyping platform for growing *Arabidopsis* in multi-well plates. **(A)** XYZ PlantScreen^TM^ growth-chamber with automatic top view RGB imaging. **(B)** RGB images of 9 DAG old *Arabidopsis* seedlings. **(C)** RGB image of an individual well-plate with the blue boundary mark created by the in-house software for analysis.

The multi-well plates with the transferred *Arabidopsis* seedlings were placed onto the OloPhen platform^1^ that uses the PlantScreen^TM^ XYZ system installed in a growth chamber with a controlled environment and cool-white LED and far-red LED lighting (Photon Systems Instruments, Brno, Czech Republic). The conditions were set to simulate a long day with a regime of at 22°C/20°C in a 16/8 h light/dark cycle, an irradiance of 120 μmol photons of PAR m^-2^ s^-1^ and a relative humidity of 60%. The PlantScreen^TM^ XYZ system consists of a robotically driven arm holding an RGB camera with customized lighting panel and growing tables with a total area of approximately 7 m^2^ with a capacity of 480 multi-well plates fixed in customized trays for accurate positioning of every plate (**Figure 1A**). The XYZ robotic arm was automatically moved above the plates to take RGB images of single plates from the top. RGB images (resolution 2500 × 2000 pixels) of a single plate with a file size of approximately 10 MB in the PNG compression format (**Figure 1B**) were stored in a database on a server, using a filename containing information about the acquisition time and the (x, y) coordinates of the camera. The data were automatically stored in PlantScreen XYZ database, exported by PlantScreen Data Analyzer software and analyzed using an in-house software routine implemented in MatLab R2015 (for details see Results). The application can be used without any charge upon obtaining a license from the author. The license can be obtained by e-mail to Palacky University upon agreeing not to use the application for commercial purpose. After obtaining the license, the end-user will be provided (free of charge) with the MCRInstaller.exe. MCRInstaller simulates the MatLab environment on computers where MatLab is not installed and enables to execute the applications. To obtain the application executable files, please contact the author Tomas Furst by email tomas.furst@upol.cz. The email must contain the following statement: “Neither the application nor the MCRInstaller will be used for any commercial purpose”.

### Statistical Analysis and Data Representation

To assess the differences between the projected areas of two or more groups of plants at a particular time-point, the non-parametric Kruskal and Wallis one-way analysis of variance by ranks was used. The test compares the medians of the samples in the respective groups, and returns a *p*-value for the null hypothesis that all samples are drawn from the same population. For the analysis of multidimensional data, visual representations in the form of box plots, histograms, and animations, created using the MatLab R2015 software, were used to capture the time dimension of the problem.

## Results

### The Assay Workflow

The presented assay was designed for automated large-scale analysis of *Arabidopsis* rosette growth *in vitro* using multi-well plates. The *in vitro* cultivation of the plants confers the advantage of precise control of the growth media and easy supplementing and dosing of tested factors. Importantly, it allows easy introduction of generalized randomized block designs (GRBDs), the statistical theory of the design of experiments that is used to study the interaction between blocks and treatments. In our method, a block is represented by a multi-well plate containing an array of plants. The plates can then be randomized within the growth area and replicated in the case of optimization of the method when a blocking factor is tested as the potential source of variability. Typically, such a source is differences in the growth chamber microclimate, however, one usually underestimated factor that introduces nuisance variables is the operator preparing the treatment.

**FIGURE 2:**
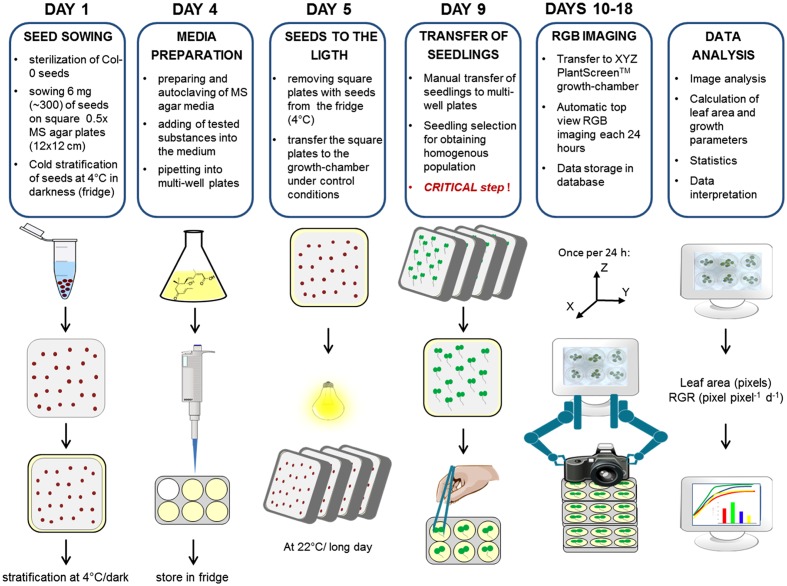
Protocol for high-throughput phenotyping for screening of *Arabidopsis* rosette growth in multi-well plates. Col-0 were germinated in 0.5× MS under long day controlled conditions. Seedlings of similar sizes were transplanted into multi-well plates with fresh MS medium 4 days after cold stratification. Plates were transferred to a XYZ PlantScreen^TM^ growth-chamber for automatic RGB imaging and data analysis.

Thus, to ensure an appropriate experimental procedure, we established the protocol schematized in **Figure 2**; this was used for optimization of the method, its validation, salt stress response and growth interaction studies. The protocol takes a total of 18 days and consists of several steps including seed sowing, cold stratification, transferring the seedlings into the multi-well plates, time-course RGB-imaging, data processing and analysis (**Figure 2**). The seeds are sown onto the square plates (12 cm × 12 cm) containing compound/stressor-free medium at a density of about one seed per 1.5 cm^2^, facilitating manipulation during subsequent seedling transfer. After cold stratification, the plates are placed vertically in the growth chamber, thus preventing growth of the root into the solid media. This minimizes any possible damage to the tiny 3 days after germination (DAG) old seedlings during the transfer into the multi-well plates (one seedling per well). The transfer of the seedlings was introduced into the protocol as an important step to achieve a method in which the effect of the tested conditions (stressors, chemicals, etc.) is scored not earlier than during the stage when the cotyledons are expanded, thus avoiding the possible effect on the process of germination. As described later in the text, the selection of seedlings of similar size for the transfer into multi-well plates represents a critical point in this method. After seedling transfer, the multi-well plates are placed into the PlantScreen^TM^ XYZ system and after about 24 h of acclimation, automated RGB-imaging is performed every day for the next 9 days. When the platform is at capacity, the whole imaging run takes 70 min, thus in theory there can be 12–13 imaging runs within the 16-h-long light period, producing a dense-point growth curve. In the case of a well optimized assay, the sensitivity of the analysis allows scoring differences in the rosette area over only 2-h-long intervals. As shown in the example of two independent 12-well plates, the average increase in the green area in 2 h is 2-3% (**Supplementary Figure S1**). This offers the possibility for further optimization to increase the assay through reduction of the entire time of the assay and/or use of multi-well plates with a larger number of smaller wells. In the presented protocol, the growth of the green area was recorded every 24 h (typically at midday) during the 9 days. The imaging data were processed using in-house software described in the following section.

### Software for Image Processing

The RGB imaging data were analyzed using an in-house software routine, examining all the files covering the entire experiment, i.e., images of up to 1000s of plates taken at many pre-defined time-points. In the first step, fish-eye correction of each image is performed. Next, the image is registered automatically so that the positions of the wells are correctly identified. For this step, blue boundary marks on the trays are used (**Figure 1C**), together with an edge detection routine. The registration step is somewhat sensitive to errors, therefore several suggestions for the correct registration are computed and returned in order of decreasing probability. The most probable registration is tried first and the image is registered and cropped to contain only the plate with no surroundings. The plants are automatically detected by thresholding the image in the HSV color space. Since we are looking for green pixels in a generally white background, the threshold need not be very intelligent, a fixed cut-off value is used separately in each of the three HSV channels and the results are combined by means of the logical operator “AND.” If any green area is detected outside the wells, the software recognizes that it made a mistake in the registration step and returns to try the registration step with another set of plausible registration parameters. When the segmentation is successful, i.e., there is no green area detected outside the wells, the green areas of all the plants are computed and a pre-view of the registered and segmented image is saved to disk. The pre-views can be reviewed manually and any remaining errors corrected. After all the images have been analyzed, a single XLSX data file is produced which contains, in each row: the name of the file, date of acquisition, (x, y) position of the camera, and subsequently the list of 12, or 24 numbers, which represent the green areas (in pixels) of the plants in the wells. The wells are numbered column-wise. On a standard PC, the processing of a single 10 MB PNG file takes approximately 10 s with most of the time spent on the fish-eye correction routine. Since the experiment itself usually lasts for days, there was no need for any speed-optimization of the MatLab routine.

For correct data handling, we must take into account the fact that the data produced by these experiments are naturally multi-dimensional. Usually, several different treatments in various concentrations are tested together with one or more controls, with the option to include different numbers of wells per plate. Thus, there are at least three independent predictors of the green area: time, type of treatment (together with its concentration), and the type of plate (6-, 12-, or 24-well). It is also important to keep track of plants that come from the same plate because it is possible that there is more correlation between the green areas of plants from the same plate than among plants from different plates. These multidimensional data are not easy to handle using standard table-processing software (e.g., Excel) because of a richer data-structure, so a table with more than two dimensions is needed. Thus, the data processing was also performed using MatLab software, creating a data structure with the same length as the number of PNG files. Each item in the structure contains the following terms: time from the beginning of the experiment, type of treatment, numerical code of the treatment, type of plate, position on the table, and a list of the green areas of the plants. The same data structure was used for the statistical analysis and data representation.

### The Assay Optimization and Validation

**FIGURE 3:**
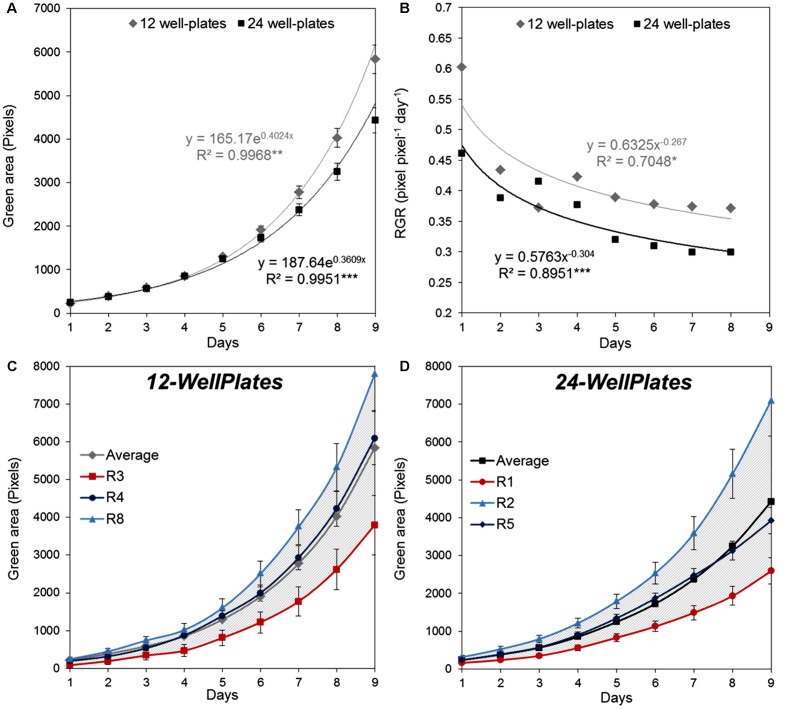
Natural variation in *Arabidopsis* rosette growth in multi-well plates under control conditions. **(A)** Green area (pixels) of 4 DAG *Arabidopsis* seedlings grown in 12-well plates (*n* = 108) or 24-well plates (*n* = 144) with 1× MS medium for 9 days. *Mean ± SE* **(B)** Relative growth ratio (RGR, pixel pixel^-1^ day^-1^) of 4 DAG *Arabidopsis* seedlings. The equations of the curves and the Pearson’s correlation coefficients with significance according to ANOVA after linearization were calculated. ^∗^*p* < 0.05; ^∗∗^*p* < 0.01; ^∗∗∗^*p* < 0.001. **(C)** Green area (pixels) of 4 DAG *Arabidopsis* seedlings’ growth in independent 12-well plates (replicates). **(D)** Green area (pixels) of 4 DAG *Arabidopsis* seedlings’ growth in independent 24-well plates (replicates). Gray striped area represents the variation between replicates compared to the average treatment effect.

The right experimental design for the assay requires introduction of a standardized protocol resulting in maximum homogeneity of the recorded trait, in this case similar plant growth on a single plate and among plate replicates. This allows application of the statistical methods to describe significance of the differences between the tested variants. To define the most suitable screening conditions to achieve HTS using *in vitro* conditions, first the response of 4-day-old *Arabidopsis* seedlings grown in 1× MS [recommended for *Arabidopsis* growth in the protocol published by Cold Spring Harbor Protocols (Recipe, 2010) and by the Arabidopsis Biological Resource Center^2^] was evaluated using a different culture plate format, with a higher number of replicates randomly distributed across the growth area. The 12- and 24-well plates were prepared following the experimental scheme (**Figure 2**) with nine and six replicates *per* variant (represented by a single plate), respectively, and the rosette size (represented by the green area) was analyzed for 9 days. The outcome of the analysis can be either a single growth curve describing the increase in green area over time (**Figure 3A**), or a curve showing the relative growth rate calculated as described Hoffmann and Poorter (2002) (**Figure 3B**). The curves for seedling growth in the 12-well and 24-well plates had similar profiles, showing that during the 9 days when data were collected, the green area of the seedling exhibits high significant exponential growth (**Figure 3A**). Although the rosette area of the seedlings grown in the 12- and 24-well plates starts to differ after 1 week of cultivation, the relative grow rate (RGR) of the seedlings shows the same tendency, with parallel curves but with higher values for the those grown in 12-well plates (**Figure 3B**). This indicates that the volume and space of the well are the main factors determining the difference observed after the sixth day.

**FIGURE 4:**
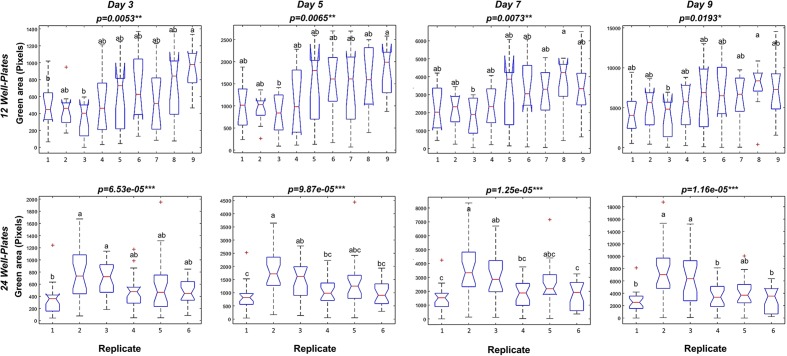
Variation between repeated well plates in *Arabidopsis* rosette growth under control conditions. Box plots representing the green area (pixels) of 4 DAG *Arabidopsis* seedlings grown in 12-well plates (9 biological replicates) or 24 well-plates (6 biological replicates) with 1× MS medium for 9 days. Different letters indicate significant differences according to Conover’s test after Kruskal–Wallis’ test. ^∗^*p* < 0.05; ^∗∗^*p* < 0.01; ^∗∗∗^*p* < 0.001.

**FIGURE 5:**
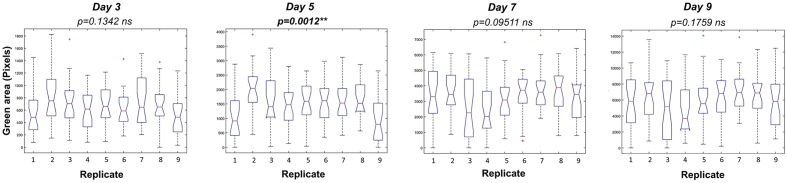
Variation among replicates in *Arabidopsis* rosette growth under control conditions after seedling selection. Box plots representing the green area of 4 DAG *Arabidopsis* seedlings grown in 24-well plates (9 biological replicates) with 1× MS medium for 9 days. Statistical analysis was performed using Kruskal–Wallis’ test ^∗∗^*p* < 0.01; ns – non-significant.

To optimize the assay we used a Kruskal–Wallis test to evaluate statistically the differences in rosette area between the plate replicates, which were randomly distributed within the growth area. Unexpectedly, significant differences in the average green areas, in some cases reaching almost 50%, were observed within the plate replicates in both types of multi-well plate at the analyzed time-points (**Figure 4**). Analysis of the rosette growth in the replicates with the smallest, intermediate and largest average growth areas (R3, R4 and R8 or R1, R2 and R5 for 12- and 24-well plates respectively) revealed similar profiles (**Figures 3C,D**), with significant differences between the two extremes (**Table 1**). Taking into account the experimental set-up of the assay, the possible reasons for the differences in the average rosette size in the randomly distributed replicates could be either different micro-climatic conditions in the growth chamber or the non-randomized selection of the seedlings at the time of transfer with respect to developmental stage, resulting from the natural heterogeneity of the population. Measurements of the micro-meteorological conditions in the phenotyping chamber did not reveal any differences. For this reason, in the next step we increased the number of germination plates to increase the population of the seedlings. This allowed us to improve our selection of the 4-day-old seedlings, ensuring that they were all a similar size at the time of transfer, discarding any particularly large or particularly small seedlings. The more careful selection of seedlings did, indeed, result in standardization of population heterogeneity between plate replicates, and almost no statistically significant differences between the average rosette areas of the nine replicates were found at the different time-points (**Figure 5**).

**Table 1:** Statistical differences among replicates of *Arabidopsis* rosettes grown under control conditions.

Well-Plate	Replicate	Day 1	Day 2	Day 3	Day 4	Day 5	Day 6	Day 7	Day 8	Day 9
12WP	R3	a	a	b	b	b	b	b	b	b
	R4	a	a	ab	ab	ab	ab	ab	ab	ab
	R8	a	a	a	a	a	a	a	a	a
24WP	R1	b	b	a	a	a	a	a	a	a
	R2	a	a	b	b	b	b	b	b	b
	R5	ab	ab	a	a	a	a	a	a	a

*Different letters indicate significant differences in green area (pixels) of 4 DAG old *Arabidopsis* grown in 1× MS medium over time for selected replicates of 12-well plates and 24-well plates according to Conover’s test after a Kruskal–Wallis test (*p* < 0.05)*.

**FIGURE 6:**
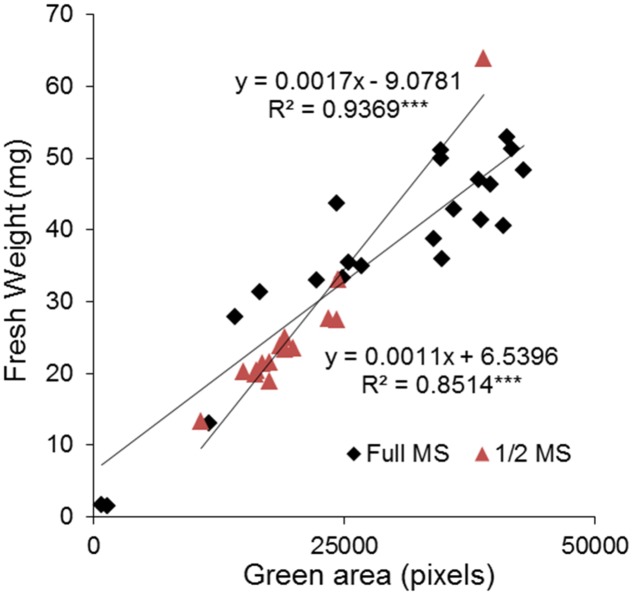
Validation between high-through phenotyping and traditional biometric methods for the analysis of *Arabidopsis* rosette growth in multi-well plates. Correlation between projected green area (pixels) and fresh weight (mg) of 13 DAG *Arabidopsis* seedlings grown in 24-well plates with 1× and 0.5× MS for 9 days. The equation of the curve and the Pearson’s correlation coefficient with significance according to ANOVA were calculated. ^∗∗∗^*p* < 0.001.

Finally, to test the reliability of the method, we compared the green area estimated by automated RGB imaging with the weight of the rosettes determined manually. The rosette of individual plants grown *in vitro* in 24-well plates containing 1× MS and 0.5× MS medium, respectively, were harvested on the last day of measurements and the fresh-weight (FW) of individual plant rosettes was determined. Subsequently, correlations between the green area and FW were calculated using Pearson’s coefficient and the significance determined after ANOVA. In both growing conditions, a highly significant correlation was obtained with correlation coefficients of 0.94 and 0.85, respectively (**Figure 6**). The relationship between green area and FW of *Arabidopsis* rosettes showed more homogeneous size of the plants grown in 0.5× MS compared to 1× MS. In 1× MS conditions the distribution of the population was broader, with higher number of smaller (<10 mg of FW) and bigger plants (>40 mg of FW), suggesting that the nutritional conditions contribute to the population phenotype.

### Standardization of the Assay for HTS of *Arabidopsis* Rosette Growth in Normal and Stressed Conditions

#### State-of-the-Art of Conditions Used for *In Vitro* Growth of *Arabidopsis*

**FIGURE 7:**
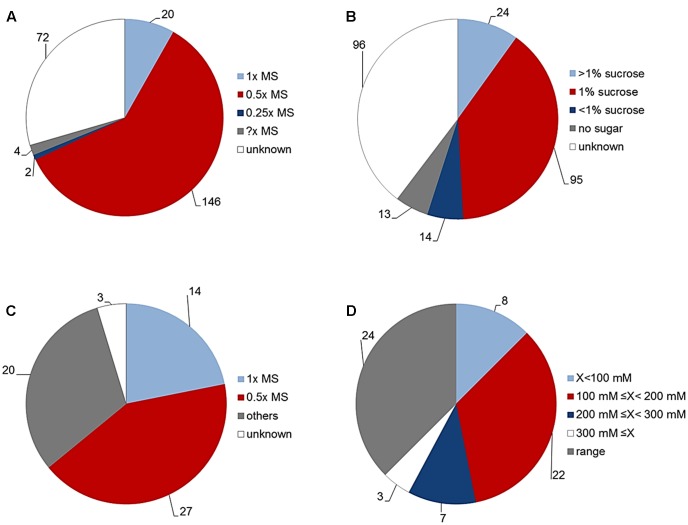
“Normal” *in vitro* growth conditions for *Arabidopsis* plants. **(A)** Culture medium used for *Arabidopsis* grown *in vitro* according to articles (*n* = 242) published in the five highest impact Plant Science journals (Web of Science) during 2016. MS = Murashige and Skoog basal salt mixture. **(B)** Concentration of sucrose added to the culture medium for *Arabidopsis* grown *in vitro* in the same publications. **(C)** Culture conditions used for salt-stress studies of *Arabidopsis* according to articles (*n* = 64) published in the five highest impact Plant Science journals (Web of Science) from 2012 to 2016. **(D)** Concentration of salt published in the same articles for stress studies of *Arabidopsis*.

To standardize our assay so that it can become a universal HTS tool suitable for analysis of *Arabidopsis* rosette growth, we first performed an in-depth literature review of the typical conditions used for *in vitro* growth of *Arabidopsis*. The main goal for the presented assay is evaluation of rosette growth (green area) under normal and stressed conditions. Thus, we tried to investigate which growth conditions are typically used by plant biologists to represent “normal” for *Arabidopsis* growth *in vitro* and which conditions are chosen to study stress responses, with the focus on salinity. To achieve this, we analyzed “materials and methods” sections of research articles published in the five research journals with the highest impact factor in the category “Plant Science” (based on the ranking of Web of Science). We attempted: (1) to find the growing medium composition used most often for *in vitro* growth of *Arabidopsis* for publications from 2016, and (2) to determine the conditions used to study its response to salinity in publications from the last 5 years (2012–2016). To determine what is meant by “normal *in vitro* conditions,” we analyzed 242 articles published during 2016. As presented in **Figures 7A,B**, a high diversity with respect to plant growth conditions was found. Approximately 70% of the studies used MS medium as a source of nutrients, with many variations in the concentration of sucrose and gelling agent. Half strength MS (0.5× MS) was used in about 60% of studies, followed by full strength MS (1× MS) in about 8% of studies and even quarter strength MS (0.25× MS) in 1% of studies. Surprisingly, in about 30% of the articles examined, information about the type of growing medium was missing (**Figure 7A**). The use of sucrose as a source of energy for *in vitro* grown *Arabidopsis* was also highly variable. About 55% of studies reported using sucrose in different concentrations, only 5% of the studies did not use sucrose in the growing medium, and surprisingly, about 40% of published articles did not specify whether sucrose was used (**Figure 7B**). Among the work that did mention the use of sucrose, the most common concentration was 1% and higher, only in 10% of the cases was a concentration less than 1% used (**Figure 7B**). Next, we analyzed the growth conditions used in the salt-response studies (**Figures 7C,D**). Of 64 articles, about 70% reported using MS medium (with a clear preference for 0.5× MS over 1× MS), while in the rest of the studies salinity was applied through hydroponics, soil, or an unspecified medium (**Figure 7C**). With respect to the concentration of salt applied, we found that 62% of the articles on the subject used only one concentration of NaCl, whilst the remaining 38% reported using a range of salt concentrations (**Figure 7D**). More than half of the studies used 100–200 mM NaCl; of the remaining studies, there were similar numbers that used concentrations either lower than 100 mM, or higher than 200 mM; and, surprisingly, in a few cases an extremely high concentration of NaCl (≥300 mM) was used (**Figure 7D**).

### Standardization of Control Conditions for the Assay

**FIGURE 8:**
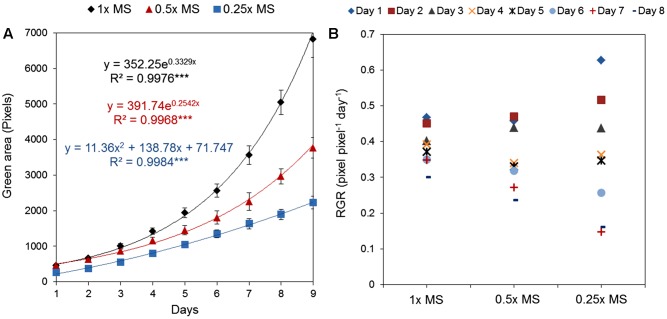
Natural variation in *Arabidopsis* rosette growth in different MS media. **(A)** Green area (pixels) of 4 DAG *Arabidopsis* seedlings grown in 12-well plates (*n* = 36) with different MS media for 9 days. *Mean ± SE*. The equations of the curves and the Pearson’s correlation coefficients with significance according to ANOVA after linearization were calculated. ^∗∗∗^*p* < 0.001. **(B)** Relative growth ratio (RGR, pixel pixel^-1^ day^-1^) of 4 DAG *Arabidopsis* seedlings.

To select our standardized normal conditions we tested experimentally whether MS concentration influenced *Arabidopsis* rosette growth and also evaluated the need for sucrose as a component of the growing medium. First, MS medium without sucrose was used in different concentrations; 0.25×, 0.5×, and 1×. A clear concentration-dependent increase in rosette area was found, indicating that 1× MS is the best growing medium for *Arabidopsis* seedlings *in vitro* (**Figure 8A**). Although the RGR of the seedlings grown on 0.5× MS was comparable to those on 1× MS during the first 4 days, in the second half of the growth period the development of these seedlings slowed and their RGR was 13% lower than for seedlings on 1× MS, decreasing to 25% during the last 2 days (**Figure 8B**). This suggests that use of lower MS concentration than 1× can result in a change from optimal to suboptimal growth conditions during the period that the experiment is running and the seedlings are inadvertently subjected to low nutrient stress during their, otherwise, exponential growth period.

As mentioned above, the use of sucrose in growth media is also generally very variable, ranging from a concentration of 0 to 3%. As discussed later, the presence of sucrose in the growing medium leads to substantial changes in the physiology of the developing seedling, conditioning seed germination and modifying plant metabolism (Ohto et al., 2001; Eckstein et al., 2012). Hence, we tested how the exogenous addition of sucrose alters the growth of *Arabidopsis* seedlings in optimal nutritional conditions and compared plant performance when grown on 1× MS medium containing 0, 0.1, and 1% sucrose. No significant differences were found in the increase of the rosette areas of the seedlings grown with and without sucrose over the duration of the experiment (**Supplementary Figure S2**). Taking into account these results, we decided to use 1× MS without sucrose as the standard growing medium for our assay.

### Use of the Assay in the Salt-Stress Studies

**FIGURE 9:**
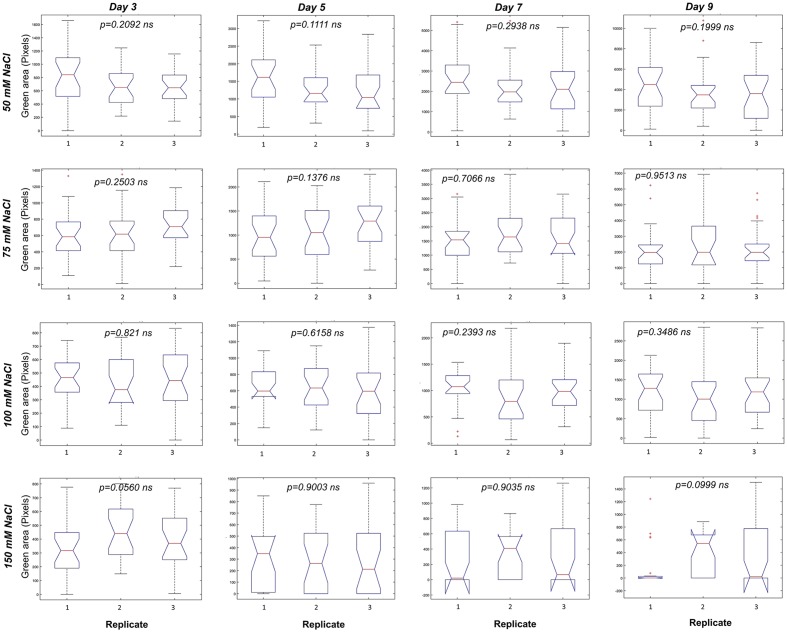
Variation among replicates in *Arabidopsis* rosette growth under salt-stress. Box plots representing the green area (pixels) of 4 DAG *Arabidopsis* seedlings grown in 24-well plates with 1× MS medium and different NaCl concentrations for 9 days. Statistical analysis was performed using Kruskal–Wallis’ test. ns, non-significant.

**FIGURE 10:**
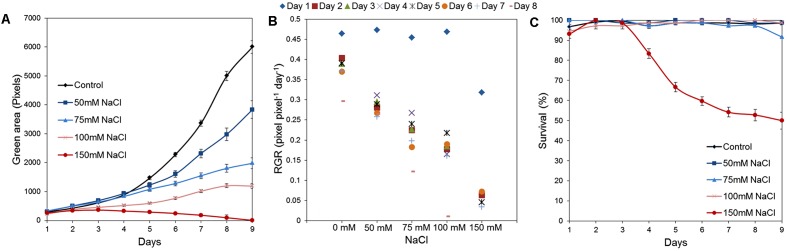
Natural variation in *Arabidopsis* rosette growth under salt-stress. **(A)** Green area (pixels) of 4 DAG *Arabidopsis* seedlings grown in 24-well plates (*n* = 48) in 1× MS with different NaCl concentrations for 9 days. *Mean ± SE* **(B)** Relative growth ratio (RGR, pixel pixel^-1^ day^-1^) of 4 DAG *Arabidopsis* seedlings. **(C)** Survival (%) of the seedlings.

Our platform has sufficient capacity to allow simultaneous testing of large numbers of variants. This can be employed for evaluation of chemical libraries and/or genetic populations in normal and stressed conditions and for cross-testing of a wide range of concentrations of stressors and/or tested compounds. To illustrate the potential of our assay to be used as a tool for large-scale stress-response studies, we performed an experiment in which the effect of salt on *Arabidopsis* rosette growth was tested using 1× MS medium supplemented with different concentrations of NaCl (50, 75, 100, and 150 mM). Three replicates of a 24-well plate were used for each tested variant, with no significant differences among them throughout the experiment (**Figure 9**). Both time-dependent increase in shoot area and RGR were found to be negatively affected by NaCl treatment in a dose-dependent manner (**Figures 10A,B**). Even after 2 days, significant differences in the rosette area were recorded between the controls and the plants grown in the presence of 100 mM and 150 mM NaCl (**Figure 10A** and **Table 2**), due to a very fast decrease in RGR: 56 and 84%, respectively (**Figure 10B**). After 5 days, significant differences in the rosette area were also apparent between the controls and the lowest salt treatments (**Table 2**). Interestingly, salt treatment modified the population distribution causing changes in the quartiles. The moderate salinity (50 mM) increased the rosette areas of the plants of the first and the third quartiles (Q1, Q3) until the fifth day, after which the salinity started to have the expected negative effect on rosette growth (**Table 2**). The severe salt-stress conditions (100 mM and 150 mM NaCl) had clear negative effects on the rosette growth and, moreover, reduced the plant size in both quartiles. The Q1 for the plants treated with 150 mM NaCl was reduced to zero on the fifth day of the salt treatment and the survival of plants in this variant reduced from 67% at day 7 to 50% at day 9 (**Figure 10C**). Overall these results proved the potential of the assay to be used as a tool for salt-stress studies.

**Table 2:** The effect of salinity on *Arabidopsis* rosette growth.

Days	Control			50 mM NaCl			75 mM NaCl			100 mM NaCl			150 mM NaCl		
	Q1	Q3		Q1	Q3		Q1	Q3		Q1	Q3		Q1	Q3	
Day 1	157.5	400.0	a	226.3	442.5	a	228.3	403.5	a	106.5	334.3	b	132.8	409.3	a
Day 2	278.0	568.5	bc	390.8	664.0	a	409.5	628.3	ab	248.8	495.0	c	234.8	554.5	c
Day 3	403.5	846.3	a	508.3	935.3	a	447.3	812.3	a	312.8	601.3	b	253.8	549.0	b
Day 4	473.5	1291.3	a	676.8	1337.5	a	528.0	1139.8	a	388.0	708.3	b	96.3	461.5	c
Day 5	894.0	2059.5	a	892.0	1773.0	ab	724.8	1441.3	b	437.0	838.0	c	0	546.8	d
Day 6	1514.5	3040.0	a	1271.0	2289.3	b	914.0	1816.5	b	554.8	1004.8	c	0	594.5	d
Day 7	2142.3	4496.5	a	1413.3	2731.0	b	1084.3	2080.5	c	597.8	1233.8	d	0	641.3	e
Day 8	2937.5	6321.0	a	1614.8	3657.8	b	1317.8	2400.0	c	645.5	1421.5	d	0	680.0	e
Day 9	3689.0	7976.0	a	1963.5	5311.8	b	1249.8	2728.5	c	604.3	1554.0	d	0	659.5	e

*The first quartile (Q1), the third quartile (Q3) and statistical differences among treatments in green area (pixels) of 4 DAG old *Arabidopsis* grown in 1× MS medium supplemented with different NaCl concentrations. Different letters indicate significant differences according to Conover’s test after a Kruskal–Wallis test (*p* < 0.05)*.

### Large Scale Testing Can Reveal Unexpected Interactions between Conditions/Treatments

**FIGURE 11:**
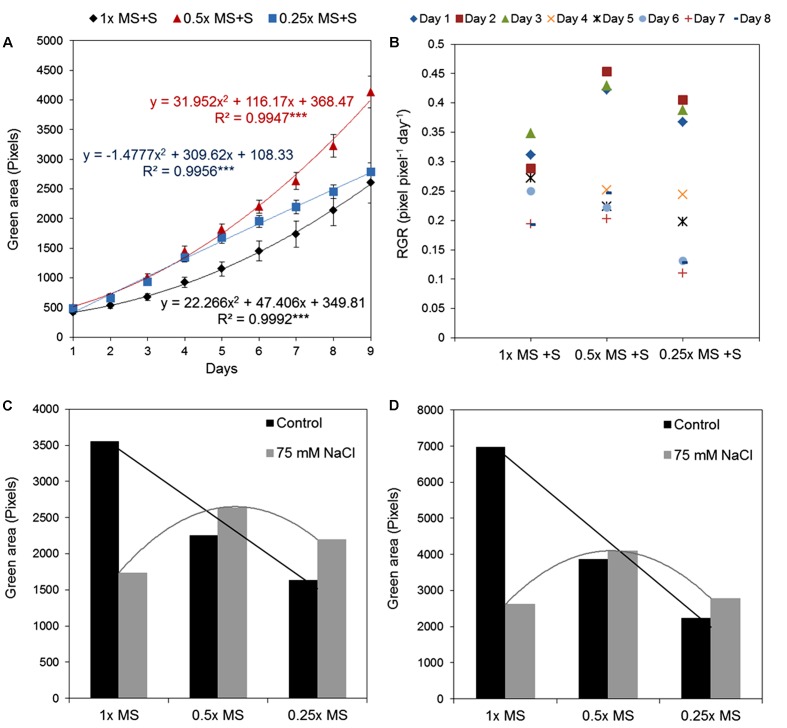
Natural variation in *Arabidopsis* rosette growth in different MS media with salt. **(A)** Green area (pixels) of 4 DAG *Arabidopsis* seedlings grown in 12-well plates (*n* = 36) with different MS media and 75 mM NaCl for 9 days. *Mean ± SE*. The equations of the curves and the Pearson’s correlation coefficients with significance according to ANOVA after linearization were calculated. ^∗∗∗^*p* < 0.001. **(B)** Relative growth ratio (RGR, pixel pixel^-1^ day^-1^) of 4 DAG *Arabidopsis* seedlings grown under the same conditions. **(C)** Comparison among 11 DAG *Arabidopsis* seedlings grown in different MS media with or without 75 mM NaCl. **(D)** Comparison among 13 DAG *Arabidopsis* seedlings grown in different MS media with or without 75 mM NaCl.

In the previous text we described the effect of different salt concentrations on the *Arabidopsis* rosette grown under optimal nutrient conditions (1× MS). As mentioned above, analysis of the typical conditions used in salt-stress studies, 0.5× MS was mostly chosen as the source of nutrients (**Figure 7C**). This fact led us to perform an experiment in which the effect of 75 mM NaCl (identified in this study as representing medium salt stress) on *Arabidopsis* rosette growth was tested in MS medium of three different strengths, i.e., 0.25×, 0.5×, and 1×. Each variant comprised three replicates on a 12-well plate and no statistical differences among them were found according to a Kruskal–Wallis test (**Supplementary Figure S3**). When the green area of the different treatments was analyzed we obtained an unexpected result: a significant interaction between MS concentration and salt treatment (**Table 3** and **Figures 11A,B**). Both time-dependent increase in the green area of the rosette and decrease in RGR were higher in salt-stressed plants grown in 0.5× MS than those in 1× MS (**Figures 11A,B**). When they were compared with the plants grown in different MS without salt (**Figure 8A**), we observed that whereas plants grown in 1× MS without salt had at least 2-4 times bigger rosettes compared to the salt stressed ones after 7 and 9 days of the treatment, respectively, no significant differences were observed between those *Arabidopsis* grown with and without 75 mM NaCl in 0.25× and 0.5× MS (**Figures 11C,D** and **Table 3**). Interestingly, whereas the salt treatment reduced the quartiles, median and average rosette area of the plants grown in 1× MS by a factor of four, whilst keeping similar minimum and maximum values, it improved the Q1 and Q3, and the minimum size of the plants grown in the low nutrient media (**Table 3**). These results were further confirmed by the population distribution of each treatment over time (**Supplementary Figure S4**), where the salt treated 0.25× and 0.5× MS variants presented a narrower distribution and more homogeneous populations with plants of similar rosette size compared to their respective controls. The analysis also revealed the same average green areas, size heterogeneities and similar distribution of the populations of the plants grown in 0.25× MS and plants grown in 1× MS with 75 mM NaCl (**Table 3**, **Figures 8**, **11**, and **Supplementary Figure S4**). These results revealed the existence of a crucial interaction between the concentration of nutrients and the salt treatment that conditions the stress response and growth capacity of the plants through the heterogeneity of the plant population.

**Table 3:** The interaction between MS concentration and salinity for *Arabidopsis* rosette growth.

Treatment	Day 7						Day 9					
	Q1	Median	Q3	minimum	Maximum	*SE*	Q1	Median	Q3	Minimum	Maximum	*SE*
1x MS	4030	5530 a	6528	386	7919	342.5	5008	7007 a	9031.75	381	11156	499.4
1x MS + Salt	9334	1981 c	2948	193	7307	257.2	1004	2043 d	3802	194	9528	339.3
0.5x MS	1924	2911 b	3785	410	7375	210.1	2440	3719 bc	4700	454	10493	289.7
0.5x MS + Salt	2362	3359 b	3890	1320	6331	191.9	2916	4139 b	5000.25	1423	7699	267.0
0.25x MS	1154	2108 c	2553	346	3785	150.3	1424	2504 d	2889.25	371	4588	175.2
0.25x MS + Salt	2059	2528 bc	2816	556	3669	115.4	2216	2631 cd	3500.5	490	4625	153.8

*The first quartile (Q1), the median, the third quartile (Q3), the minimum (min.), the maximum (Max.) green area (pixels) of 4 DAG old *Arabidopsis* after 7 and 9 days grown in different MS media supplemented with 75 mM NaCl. Different letters indicate significant differences according to Conover’s test after a Kruskal–Wallis test (*p* < 0.05)*.

## Discussion

Recent advances in high-throughput phenotyping allow simultaneous screening of multiple quantitative traits of plant growth under different environmental conditions (Humplík et al., 2015a; Rahaman et al., 2015). However, many of the existing phenotyping systems still have a limited capacity to measure a large number of plants in a short time. For example, the GROWSCREEN FLUORO system can measure 30 plants per run, and after each run the plates must be manually exchanged (Jansen et al., 2009). Other published protocols have improved the total throughput to 200 plants (Awlia et al., 2016), or even 800 plants per hour (Arvidsson et al., 2011), however, actual throughput is in fact defined by the number of variants being tested, the number of replicates, and the number of plants per variant/replicate. For HTS approaches, transferring to *in vitro* conditions allows the miniaturization of the bioassay and an increase in the number of both variants and replicates. One example is the new work recently published for HTS of 1000s of compounds with growth regulator activity using *Arabidopsis* grown *in vitro* in 24-well plates (Rodriguez-Furlán et al., 2016). However, in this method, it takes 20 min per plate for image analysis using a scanner, which defines the number of variants and plates that can be used as replicates. It is important to mention here that 20 min per plate or series, especially in plant species like *Arabidopsis* with short life cycles and very fast growth, can be problematic. In our study we observed changes in the size of the green area of 8-day-old plants in periods as short as 2 h (**Supplementary Figure S1**). Thus, the long intervals associated with semi-automated systems that have slow image acquisition can introduce significant bias. Similar limitations can be detected in the recently published rosettR method, where the image acquisition is performed by microscope and the plates are changed manually (Tomé et al., 2017). Thus, our main goal was to develop a fast, robust and reproducible high-throughput *in vitro* bioassay for *Arabidopsis*. Our system delivers the advantage of fast fully automated measurements of the rosette growth of 11,000 *Arabidopsis* plants in less than 2 h, allowing a simultaneous study of different growth conditions without compromising the number of variants, replicates and plants per treatment, as summarized in **Table 4**. To achieve this, fully automated image-processing software and data analysis for evaluating the reproducibility of *in vitro* growth conditions using *Arabidopsis* as the plant material was developed and various growing conditions and experimental set-ups were tested. The optimal growing conditions for *Arabidopsis in vitro* growth in different well format plates (12-well and 24-well plates) were full MS + 0.6% agar without sucrose. In preliminary tests, we detected significant differences between replicates over time in both types of well plate used (**Figure 2**). After several testing runs we identified the preliminary selection of the plant seedlings for transplantation into the multi-well plates as the main influencing factor within the bioassay, significantly affecting reproducibility. Indeed, the way that germination timing influences phenotypic expression post-germination in *Arabidopsis* (Donohue, 2002), and affects plant survival (Joosen et al., 2012) have been described previously. Thus, careful selection of the plants to be used in the experiment has been highlighted and implemented in some research, ensuring synchronization of plant germination and then selecting seedlings that germinated at the same time and/or are at the same developmental stage (Humplík et al., 2015b; Awlia et al., 2016). In a recent study, specific software was presented for this purpose (Clauw et al., 2015). In our work, the selection of similar 4-day-old *Arabidopsis* all at the same developmental stage allowed us to obtain a reproducible methodology for growing, avoiding significant differences between replicates used in the different treatments (**Figures 4**, **6**). In addition, our method also conserved population heterogeneity over time, thus permitting rapid identification of the differences in rosette area among phenotypes (**Supplementary Figure S2**).

**Table 4:** The capacity of the high-throughput *in vitro Arabidopsis* bioassay using different well plates.

Type of well plate	No. plants	Replicates	Platform capacity	Total plants	No. variants	Assay duration
6-Well Plates	6	3		2880	160	14 days
12-Well Plates	12	2	480 Plates	5760	240	9 days
24-Well plates	24	1		11520	480	9 days

After optimization of the standard conditions for the *Arabidopsis* HTS, we further optimized the methodology for evaluating plant response to stress. As an example of a form of stress condition, we examined the effect of salinity on plant growth. Salinity is the main environmental factor responsible for decreasing crop productivity, affecting more than 20% of the cultivated land worldwide (Gupta and Huang, 2014). Salt stress affects plant growth in two phases: the first and rapid osmotic phase that inhibits growth of young leaves, and the second and slower ionic phase that accelerates senescence of mature leaves. In the osmotic phase, which starts immediately after the salt concentration around the roots increases to a threshold level (around 40 mM NaCl for most plants or less for sensitive plants like rice and *Arabidopsis*), the rate of shoot growth decreases significantly (Munns and Tester, 2008). The second, ion-specific, phase of plant response starts when salt accumulates to toxic levels in the source leaves, which rapidly die. This last phase dominates in high salinity conditions or in sensitive species. In our work we have demonstrated that the growth of a sensitive species such as *Arabidopsis* is not so highly affected by salt stress of 40 mM and the growth inhibition is only apparent over the time. On the other hand, the plants grown in salt concentrations of 150 mM showed very dramatic growth inhibition and fast senescence (yellow tissues) leading to death (**Figure 10**), most probably because of reaching the second salinity phase. Similar plant survival was obtained in *Arabidopsis* ecotype Col-0 grown *in vitro* using MS medium and 150 mM NaCl after 4 days [≅ 15% (Zhao et al., 2013)] or 10 days [≅ 50% (Feng et al., 2015)] of exposure. Interestingly, similar curves for plant growth over time were also presented in a recently published HTS technique for studying salinity tolerance in *Arabidopsis* using soil, where 50 mM NaCl did not significantly affect the growth until 8 days of exposure, but very rapid growth inhibition and chlorosis in plants was indiced when 150 mM NaCl was applied (Awlia et al., 2016).

Surprisingly, in our experiments we also observed that salt-induced growth inhibition and *Arabidopsis* tolerance capacity are defined by an interaction between salt and the nutrient concentrations in the growing medium. Higher tolerance to salinity was found in plants grown in the 0.5× MS medium than those ones grown in 0.25× or 1× MS (**Figure 11** and **Table 3**), and a higher number of dead and smaller plants was observed for the combination 1× MS and salinity (see **Supplementary Figure S4**). These results suggest that some specific nutrients may be responsible for plant sensitivity to stress and that a reduction in their concentration could delay the senescence effect characteristic of the second phase of the stress response, even in a sensitive species as *Arabidopsis*. It is known that salinity affects nutrient uptake in plants and induces some nutrient deficiencies, such as a reduction of Ca^2+^, N, and K levels in different plant species (Pérez-Alfocea et al., 1996; Gunes et al., 2007; Koksal et al., 2016). Tuna et al. (2007) studied the effect of nutrient supplementation with CaSO_4_ for mitigating salt stress-induced losses in crop production. They showed that tomato plants exposed to 5 mM CaSO_4_ exhibited improved salt tolerance, increasing the concentration of specific ions in the plant such as K^+^, Ca^2+^, and N and reducing the levels of Na^+^. In accordance with this, it has been shown that Ca^2+^ can move very rapidly through the plant and activates a rapid plant response to stress (Choi et al., 2014). Ca^2+^ is one of the main nutrients present in MS medium together with N. Due to the fact that salinity greatly affects the activity of many enzymes involved in the N metabolism (Dubey, 1997), we propose that plants growing in 1× MS + salt are absorbing too many nutrients and exceeding their assimilation capacity under these conditions. However, more studies are needed to corroborate this suggestion. This might also explain the discrepancies in some published works using *Arabidopsis* plants grown in 0.5× MS medium with high salt concentrations (Pitzschke et al., 2014; Wang et al., 2015; Dolata et al., 2016). To clarify these results, more simultaneous studies of these two variables (salinity and nutrients) are needed. In addition, to avoid controversial results and to ensure that different studies are comparable, we think there is a need for a standard global protocol, specifying the *in vitro* growing conditions for *Arabidopsis*.

## Conclusion

In this work we present a highly reproducible *in vitro* HTS assay using *Arabidopsis* that offers simplified scoring of phenotypes and permits large-scale miniaturized screening over a short time, allowing faster identification of phenotypes with different tolerances and the evaluation of possible candidate molecules that can offer a simple solution to the production problems caused by salinity.

## Author Contributions

NDD, TF, JH, and LS designed the experiments and performed the data analysis. LU and KP performed the experiments. NDD and LS supervised the study and the concept of the project. All authors discussed the results. NDD, TF, and LS wrote the manuscript.

## Conflict of Interest Statement

The authors declare that the research was conducted in the absence of any commercial or financial relationships that could be construed as a potential conflict of interest.

## Footnotes

1 http://www.plant-phenotyping.org/db_infrastructure#/tool/57

2 https://abrc.osu.edu/seed-handling

## Supplementary Material

FIGURE S1 Green area (pixels) of 9 DAG *Arabidopsis* seedlings grown in independent 12-well plates (replicates, R1 and R2) at 12:00 and at 14:00 H.

FIGURE S2 Green area (pixels) of 4 DAG *Arabidopsis* seedlings grown in 12-well plates (n = 36) with 1× MS medium with different sucrose concentrations (0, 0.1 or 1%) for 8 days. *Mean ± SE.*

FIGURE S3 Variation among replicates in *Arabidopsis* rosette growth in different MS media with salt. Box plots representing the green area (pixels) of 4 DAG *Arabidopsis* seedlings grown in 12-well plates containing different MS media with or without 75 mM NaCl for 9 days. Statistical analysis was performed using Kruskal–Wallis’ test. ^∗^*p* < 0.05; ns, non-significant.

FIGURE S4 Distribution of population heterogeneity in *Arabidopsis* rosette area grown in different MS media with or without 75 mM NaCl for 7 days.
