# Supplementary figures and images for: An Automated Method for High-Throughput Screening of Arabidopsis Rosette Growth in Multi-Well Plates and Its Validation in Stress Conditions
Source: Front Plant Sci. 2017 Oct 4;8:1702. doi: 10.3389/fpls.2017.01702 (PMC5632805; doi:10.3389/fpls.2017.01702)

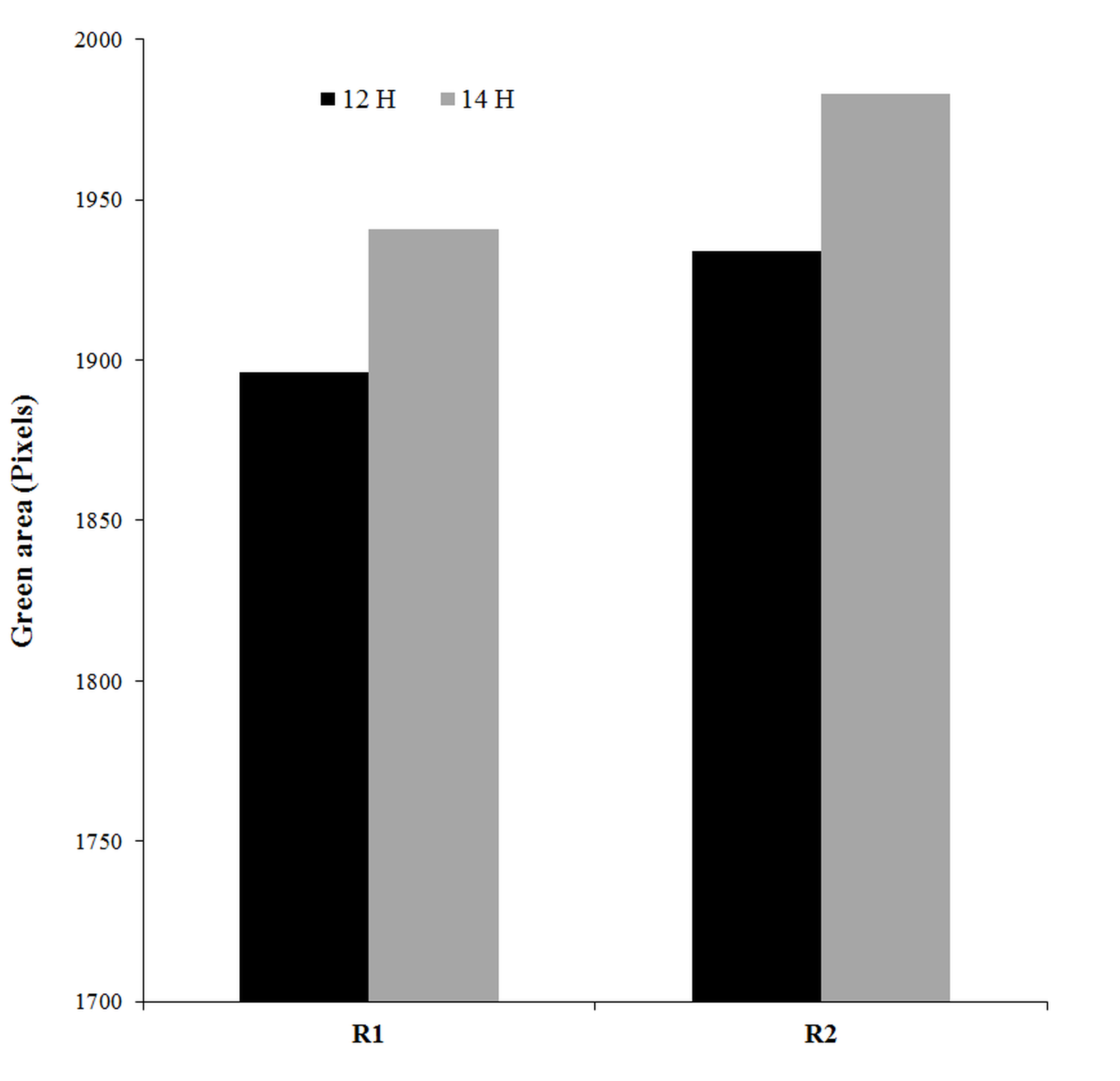

Supplement: FIGURE S1 — Green area (pixels) of 9 DAG Arabidopsis seedlings grown in independent 12-well plates (replicates, R1 and R2) at 12:00 and at 14:00 H. [file Image_1.TIF]

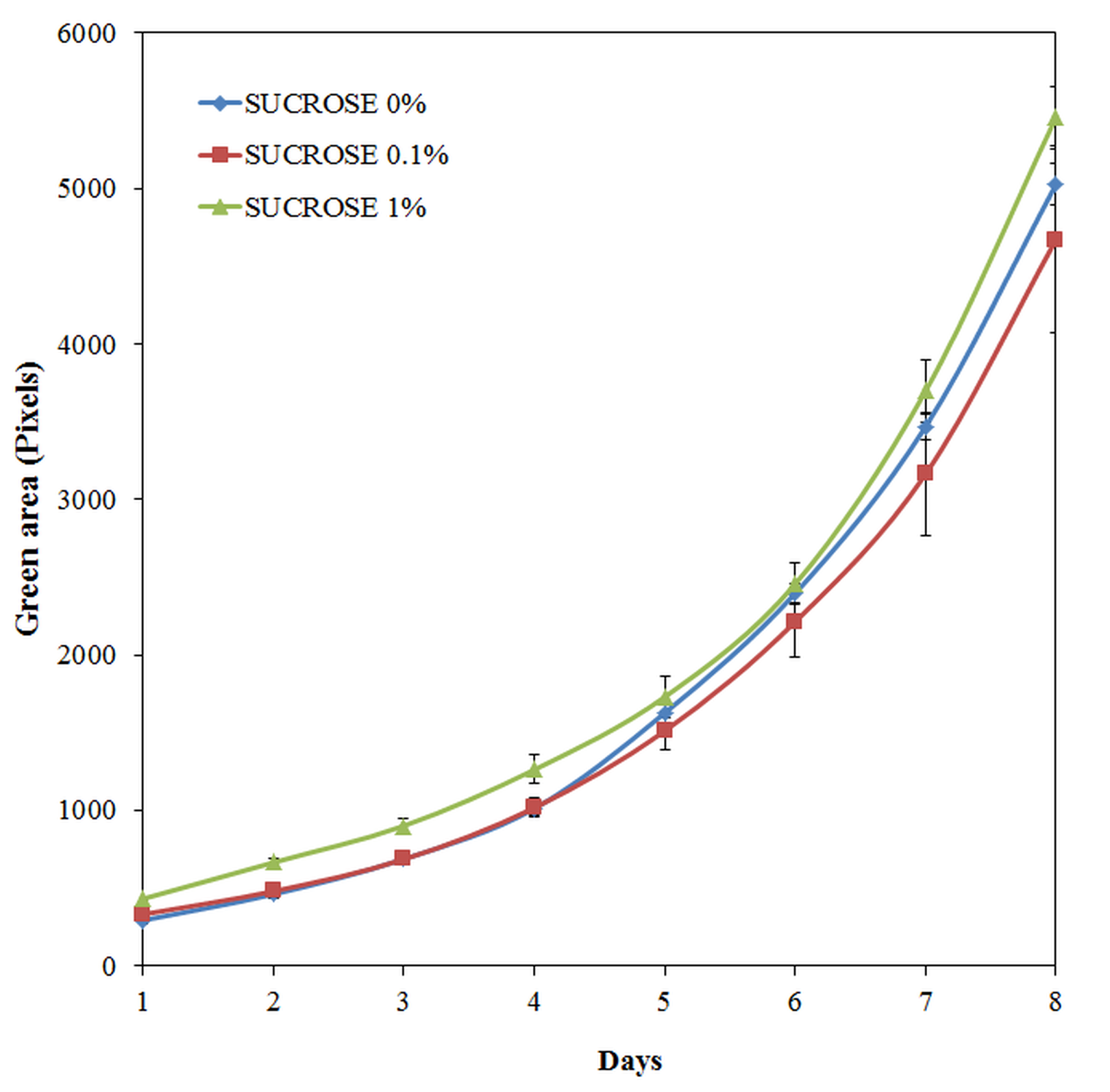

Supplement: FIGURE S2 — Green area (pixels) of 4 DAG Arabidopsis seedlings grown in 12-well plates (n = 36) with 1× MS medium with different sucrose concentrations (0, 0.1 or 1%) for 8 days. Mean ± SE. [file Image_2.TIF]

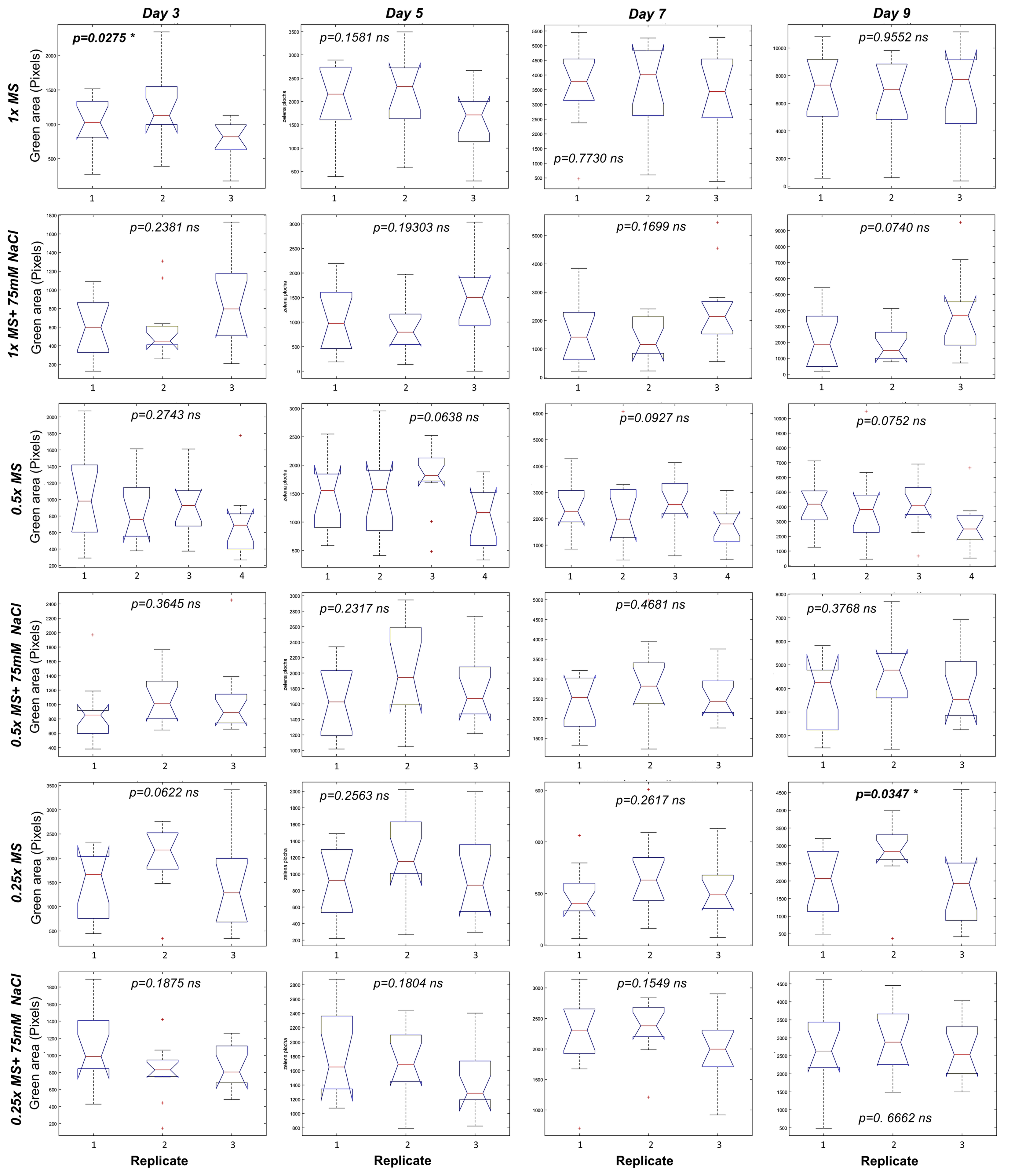

Supplement: FIGURE S3 — Variation among replicates in Arabidopsis rosette growth in different MS media with salt. Box plots representing the green area (pixels) of 4 DAG Arabidopsis seedlings grown in 12-well plates containing different MS media with or without 75 mM NaCl for 9 days. Statistical analysis was performed using Kruskal–Wallis’ test. ∗p < 0.05; ns, non-significant. [file Image_3.TIF]

## Slide 1
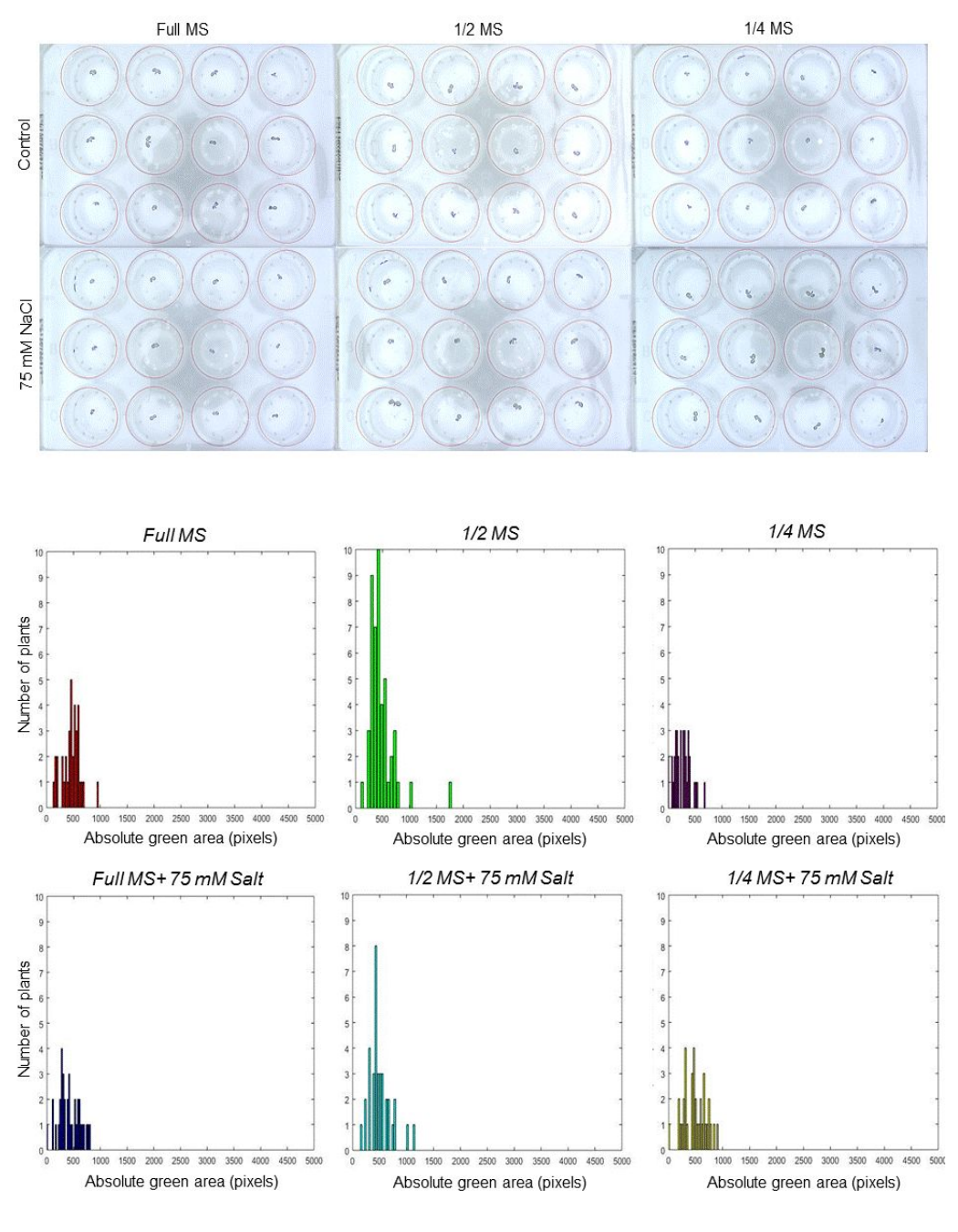

Supplement: FIGURE S4 — Distribution of population heterogeneity in Arabidopsis rosette area grown in different MS media with or without 75 mM NaCl for 7 days. [file Presentation_1.PPTX]
